# Overview of multimorbidity research in India: a scoping review protocol

**DOI:** 10.12688/wellcomeopenres.21378.2

**Published:** 2024-12-16

**Authors:** Parul Puri, Siaa Girotra, Arpita Ghosh

**Affiliations:** 1 The George Institute for Global Health India, New Delhi, New Delhi, 110025, India; 2 University of New South Wales, Sydney, New South Wales, Australia; 3 Prasanna School of Public Health, Manipal Academy of Higher Education, Manipal, India

**Keywords:** Co-morbidity, India, long-term conditions, multimorbidity, scoping review

## Abstract

**Background:**

Due to demographic and epidemiological shifts, people are living until older ages with more morbidities. These morbidities often have shared pathophysiology, which leads to a rise in coexisting health issues known as 'multimorbidity'. Primary care studies and disease burden surveys have multiplied, unveiling varied aspects of multimorbidity, yet with inconsistent definitions and methods. This protocol aims to guide an in-depth and comprehensive exploration of multimorbidity research in India through a scoping review, to understand the extent, range, and nature of research on multimorbidity in India.

**Methods:**

This study will comprehensively search the PubMed/Medline, Cochrane, and Embase databases employing a well-defined strategy. All studies published in English will be considered, provided the focus is multimorbidity and there is information specifically from India. Two reviewers will independently screen the search outcomes, and data extraction will include multimorbidity definitions, data and methods, patterns, risk factors and outcomes. The research will follow the Joanna Briggs Institute framework and adhere to PRISMA-P 2015 guidelines for reporting. Descriptive statistics and narrative synthesis will be used to summarize findings.

**Conclusions:**

Findings from this review will shed light on the extent and nature of multimorbidity research in India and help guide future research.

## Plain Language Summary

This study aims to thoroughly review existing research on multimorbidity in India. It will search major medical databases for relevant studies, focusing on those published in English and specific to India. Two reviewers will independently screen the results and extract data on definitions, methods, patterns, risk factors, and outcomes of multimorbidity. The study will follow established frameworks for research and reporting. By summarizing the findings, this review aims to inform healthcare planning, evidence-based decisions, and policymaking in India's changing health landscape.

## Background

India has witnessed significant shifts in its population demographics and health trends, driven by demographic and epidemiological transitions ^1^. These changes have resulted in a larger proportion of individuals reaching older ages, influenced by improvements in healthcare infrastructure, including advancements in medical technology and access to healthcare services. Moreover, as people migrate to urban centres in pursuit of employment, economic security, and educational opportunities, they adopt fast-paced lifestyles that leave little time for physical activity. This lack of physical activity combined with increased consumption of packaged food, coupled with environmental factors such as climate change and air pollution, as well as habits like smoking and alcohol consumption, has contributed to a rising burden of multiple morbidities. Many of these health issues are interconnected due to shared risk factors. Consequently, there has been a significant increase in the prevalence of multimorbidity – the coexistence of multiple chronic conditions within an individual ^1, 2^. Even though the terms multimorbidity and comorbidity are used interchangeably, they are related but different concepts – while both refer to people living with multiple chronic conditions, comorbidity refers to conditions in addition to an index condition, while multimorbidity is co-occurrence of two or more chronic conditions with no condition being prioritised over any other ^3, 4^.

Data on multimorbidity in India are derived primarily from health surveys and few primary studies in communities and healthcare settings. However, there is a lack of standardization in definitions, measurement tools, and reporting practices, leading to inconsistencies and challenges in synthesising findings across studies ^5– 9^. There are systematic reviews that have examined multimorbidity in low- and middle-income countries (LMICs) ^10– 15^. In these reviews, the focus is on providing a burden estimate and describing patterns of occurrence. Although, different definitions, conditions included in the definition, and data sources are discussed to explain the heterogeneity in estimates and observed patterns, the reviews do not include details about types of conditions included, rationale behind inclusion and exclusion of conditions, how conditions are defined and measured and if any theoretical framework has been used to study risk factors and outcomes. Also, it is not clear if different data sources such as prescriptions, hospital discharge summary, insurance billing data, and lab investigations have been used in addition to diagnoses to study multimorbidity. These challenges, although common among LMICs, may vary between countries at different stages of multimorbidity research and data maturity. To understand how multimorbidity research in India can be strengthened, evidence specific to India is important. However, from the reviews in LMICs it is either difficult to gather information specific to India or the information is not comprehensive or up to date. A scoping review focusing on multimorbidity research in India will be able to fill this gap by systematically mapping the available evidence, thereby facilitating more robust and comparable findings in the future.

This protocol is devised to serve as a guide for conducting the scoping review. Through the scoping review, we aim to identify and map the current research landscape related to multimorbidity in India and provide a comprehensive overview of methods used in conducting this research. Furthermore, this review will aid in identifying limitations and gaps in the evidence. We hope this review will aid multimorbidity researchers in understanding the approaches used so far and build upon it to support further development in this area. While the focus of this scoping review is on India, many of the topics discussed here may be applicable for other LMICs and for subpopulations in high-income countries.

## Methods

### Ethics and dissemination

This scoping review will involve collating already existing publicly available studies. Thus, no ethical approval is needed. We plan on disseminating the findings through presentations at seminar/conference(s) and in peer-reviewed journals.

## Review design and framework

The scoping review will follow the methodological framework adapted from the guidelines developed by Arksey and O’Malley ^16^ and refined by Levac *et al.* ^17^ and Peters *et al.* ^18, 19^. The framework suggests five steps to guide the review process: (a) defining the research question(s); (b) identifying relevant studies; (c) screening and selecting evidence; (d) charting data; and (e) collating, summarizing, and reporting the results ^20^.

### 1. Defining the research question and objective

This first step will outline the path for the scoping process. The objective of the scoping review is to understand the nature and extent of multimorbidity research in India. The specific questions we hope to find answers to are as follows:

a)   how has multimorbidity been defined and operationalized in research studies in India? Specifically, how many, and what conditions have been included, and what are the concepts behind selecting the conditions?
b)   what data have been used, how have the conditions been defined (symptoms, self-reported, medical records, etc.) and what is the sample size?
c)   in which populations has multimorbidity been studied in India?
d)   how has multimorbidity been quantified (count, indices, severity score, dyads or triads, clusters, etc.) or described (physical-mental, complex, cardiometabolic multimorbidity, etc.)?
e)   how have people with multimorbidity been characterized in terms of burden, risk factors (for example, socioeconomic status, behavioural risk factors) and outcomes (for example, hospitalization, mortality, health expenditure)?

### 2. Identifying relevant studies

***2.1 Eligibility and definition of primary concepts***

Studies will be selected based on eligibility criteria formulated using the *Joanna Briggs Institute* (2015) framework for conducting a scoping review ^21^. PCC (Population (or participants)/Concept/Context) framework, outlined below, was used to identify the primary elements of our research questions and to inform our search strategy:

**Population**

All peer-reviewed, original full-text research of any study design (observational including cross-sectional and cohort, and interventional) with participants in any age range from India or its states/union territories, having multiple long-term conditions, will be included. Both children and adults will be considered and information on multimorbidity in children – conceptualization, definition, measurement, risk factors and outcomes – will be sought from studies including children and will be reported separately. An initial search suggested multimorbidity studies looking at interventions may not yet exist in India. Hence, they are not included in objectives and are outside the scope of this review. However, if interventional studies are identified, data relevant to the stated objectives will be extracted.

**Concepts**

All studies with a primary focus on multimorbidity will be included. Although the term ‘multimorbidity’ has been in use since 1976, more recently the definition has been revised and the distinction between comorbidity and multimorbidity has been highlighted ^3, 4^. We will include studies on comorbidity, that is ones examining co-occurring multiple long-term conditions in people living with an index condition and hence will be using both the terms ‘multimorbidity’ and ‘comorbidity’ during search. We will also include similar terms like ‘polymorbidity’, 'multiple long-term condition(s), 'multiple chronic condition(s), 'multiple chronic disease(s)’ and 'multiple condition(s)'.

**Context**

We will incorporate studies carried out in community or healthcare settings within India or any of its states/union territories. There will be no limitations regarding the definition or measurement methods of multimorbidity used. The studies should encompass the definitions and methodologies employed to investigate multimorbidity, including its burden, prevalent disease profiles, risk factors, and outcomes.

***2.2 Exclusion criteria***

We will exclude the following studies:

- Studies where multimorbidity is not the central theme, for example, focus is on elderly population or frailty without consideration or reporting of multimorbidity or multimorbidity is simply reported as one of the risk factors without providing any details regarding its definition or measurement.
- Studies examining only few, selected diseases such as studies of people with diabetes, hypertension and depression.
- Multi-country studies on multimorbidity where findings from India are not separately presented.
- Published in gray empirical sources (e.g., books, dissertations, etc.), conference abstracts, and non-empirical sources (e.g., opinion papers, guidelines, policy documents, and editorials).
- Not conducted in India or any of its state or union territory.
- Published in a language other than English.

***2.3 Search strategy***

The research will undertake a systematic exploration of the databases PubMed/Medline, EMBASE (Ovid), and Cochrane Library (Ovid). Utilizing a comprehensive search strategy, both Medical Subject Headings (MeSH) and free-text keywords will be incorporated to ensure a thorough search process tailored to each database's specifications. The proposed keywords to be used in syntax search is presented in Table 1. Following the identification of articles meeting the predetermined selection criteria, a secondary screening of the references cited within these articles will be conducted to identify additional relevant literature.

**Table 1.** Proposed keywords to be used in the search syntax.

Multimorbidity		India
To be combined with OR	**To be combined with AND**	To be combined with OR
multimorbid* multi-morbid* 'co-morbid*' comorbid* 'poly-morbid*' polymorbid* 'multiple long-term condition*' 'multiple long term condition*' 'multiple chronic condition*' 'multiple chronic disease*' 'multiple condition*' 'multiple disease*		Andaman or Nicobar ‘Andhra Pradesh' ‘Arunachal Pradesh’ Assam Bihar Chandigarh Chhattisgarh ‘Dadra and Nagar Haveli’ ‘Daman and Diu’ Delhi Goa Gujarat Haryana ‘Himachal Pradesh’ ‘Jammu and Kashmir’ Jharkhand Karnataka Kerala Lakshadweep ‘Madhya Pradesh’ Maharashtra Manipur Meghalaya Mizoram Nagaland Orissa Odisha Pondicherry Puducherry Punjab Rajasthan Sikkim ‘Tamil Nadu’ Telangana Tripura ‘Uttar Pradesh’ Uttarakhand ‘West Bengal’ India

The retrieved search outcomes will be consolidated and managed using EndNote 20, a recognized citation management tool. Within EndNote 20, a meticulous deduplication process will be executed to eliminate any duplicate records and ensure the integrity of the dataset. Subsequently, the refined records will be imported into Covidence, a web-based platform designed for systematic literature reviews. Covidence will facilitate independent screening by multiple reviewers and provide a mechanism for resolving any disagreements that may arise among the reviewers during the screening process.

### 3. Evidence screening and selection

After deduplication, the articles will undergo a two-stage inclusion screening process.

***Stage 1:*** Screening will comprise of reviewing study title and abstract against the predefined inclusion and exclusion criteria.

***Stage 2:*** Once the entire list is screened, the list of articles included will be taken up for a second stage screening. In the second stage, the full text will be reviewed against the inclusion and exclusion criteria.

In both stages, two reviewers will independently review the evidence and decide whether to include a specific article. The screened records will be classified into three categories: 'no', 'maybe', or 'yes'. All articles that achieve consensus will be either included or excluded, while the decision on the others will be made after discussing with the research team. Finally, the entire screening strategy, including the number of articles included/excluded in each stage, will be presented as a PRISMA flow chart.

### 4. Data charting

A structured data charting form will be developed in MS Excel to ensure methodical data extraction. The form will be created using Excel for seamless conversion into a format suitable for subsequent data analysis. This will be done in three phases: preparation, organizing and presentation of data. We will use a mixed approach to prepare the data charting tool. For the objective (a) compiling commonly used definitions, measures used, a deductive approach is thought suitable. Whereas, for (b) mapping frequently occurring multimorbidity patterns/clusters and (c) mapping presence of risk factors, outcomes, treatment, and interventions linked with multimorbidity an inductive approach will be used. A preliminary trial of the data charting form will involve 10 studies, followed by a team meeting to assess its alignment with the research question and review objectives. The data charting form will capture various study details, but it will be subject to iterative refinements post the pilot phase and during the actual data extraction process. The pilot data charting tool is presented in Table 2.

**Table 2.** Pilot data extraction form.

*Domain*	*Data Item*	*Description*
***Bibliographic*** *** Information***	Title	Study title
	Authors	Authors
	Affiliation	Affiliation of the authors
	Publication Year	Year of publication
	Source	Journal name and study URL
***Study Purpose***	Study Objective	Objective mentioned in the study
***Study Characteristics***	Data source	Secondary/routinely collected data/hospital records Primary
	If secondary, specify	includes WHO Sage, NFHS, NSS, LASI, BKPAI, IHDS, DLHS, AHS, Others
	Year of Data	The data used in study pertains to which year/period
	Study Participants and Characteristics	Description on who are the participants: age, sex, background characteristics including socioeconomic groups (for example, urban poor), place of residence (for example, slum dwellers), social groups based on religion and caste
	Nationally Representative Data	Is the study based on a nationally representative dataset? yes/no
	Study Design	Cross-sectional/ cohort (includes registry)/interventional
	Sampling methods	Simple random/ Stratified/ Quota/ Systematic/ Cluster/ Convenience/ Snowball/ Purposive/ Stratified/ Multistage sampling design/Not Applicable
	Sample size of the study	It will include sample size (number of participants) in the study. We will mention the sample from India, when dealing with multi-country studies.
***Population *** ***Demographic*** *** Distribution***	Age range	Provide basic idea on age, sex, and place of residence (urban, rural) distribution
	Sex	Mention % female in the study
	Place of residence	Mention % rural in the study
***Contextual *** ***Information***	Study Setting	Community Hospital Based
	Multi-country	Yes/no
	Geographical Location	Name of country mention India if nationally representative), state, region or district where the study is set
	What has been studied in relation to Multimorbidity	Burden Risk factors Outcomes
***Multimorbidity *** ***Measurement***	Definition of multimorbidity used	Two or more Three or more Other Definitions including complex multimorbidity (two or more conditions affecting two or more body systems /three or more conditions affecting three or more body systems)
	Names of Conditions included	List out the names of the conditions
	Operational definition	How were the included conditions measured/operationalised?
	Number of conditions included	Exact number of conditions used to define multimorbidity
	Type of conditions included	Long-term (Chronic) Short-term (Acute) How is chronicity defined and operationalized? How is the distinction made between chronic and acute conditions?
	Nature of conditions included	▪ Acute condition (yes/no) ▪ Non-communicable (yes/no) ▪ Chronic Infections (yes/no) ▪ Mental health conditions (yes/no) ▪ Clinical risk factors such as overweight/obesity, high-risk waist-hip ratio, high cholesterol, etc. (yes/no) ▪ Symptoms that are not medical diagnoses such as fatigue, headache, nausea, dizziness, joint pain, etc. (yes/no) ▪ Health behaviours such as smoking, alcohol consumption, etc. (yes/no)
	Method of Ascertaining Chronic Condition	▪ Self-report ▪ Self-report verified by prescriptions/test reports ▪ Clinical examination/diagnosis ▪ Physician diagnosis ▪ Symptom-based assessment
	Weather they have includes information on duration of the disease	Yes/No
	Any specification on inclusion criteria for diseases included	▪ Co-morbidities of any specific index disease ▪ Specific prevalence cut-off
	Diagnosis Classification Tool	ICD-codes with round (9, 10, 11) or others
	Measures of Multimorbidity	▪ Count ▪ Index ▪ Severity
	Method used for pattern description*	▪ Latent Class Analysis ▪ Factor analysis ▪ Principal Component Analysis ▪ Cluster Analysis
	Criteria to be determined of extracted number pattern*	▪ Average silhouette method ▪ Fit indices etc
	Disease Profile/Patterns	Explain disease profile, most prevalent disease conditions and combinations. List out identified patterns and mention exactly what diseases are included in the listed patterns
***Correlates studied***	Risk Factors studied	▪ Names of the risk factors studied: Demographic (age, sex, place of residence), Socioeconomic (wealth quintile, education), Lifestyle and behavioural (smoking/ tobacco use, alcohol consumption, diet/nutrition, physical activity), Sociocultural (religion, caste/ social group, marital status, living arrangements), ▪ Any used framework or equity angle to identify the risk factors.
	Any framework used or equity angle to identify the risk factors	Yes/No
	Multimorbidity Outcomes	▪ Outcomes linked with Multimorbidity: Subjective well-being (SRH/life satisfaction), mental health outcomes, healthcare utilization, Economic burden (OOPE and catastrophic health expenses), Health related quality of life, mortality/survival, and others (specify).
	Tools used to identify outcomes	▪ Yes/No ▪ What tools were used to identify the outcomes.
***Study Limitations***		Study limitations are mentioned by the author of the manuscript.

### 5. Collating, summarizing, and reporting the results

The study will include a descriptive analysis of the bibliographic information. This will be supplemented by a qualitative content analysis of the existing literature. The synthesis will include information on commonly used definitions, measure and methodologies used, mapping frequently occurring disease profiles, risk factors, outcomes, treatment, and interventions linked with multimorbidity. Data visualization will be done to present the study findings.

## Study status

The review has commenced.

## Discussion

Multimorbidity is a growing concern in India as people are living longer with multiple long-term conditions and experiencing unfavourable health outcomes. This changing health scenario exerts significant pressure on the already-constrained healthcare systems, making it challenging to optimally allocate resources for complex management. Addressing this issue will require knowledge of conditions that cluster together and outcomes associated with them. This review is designed to understand the nature and scope of multimorbidity research in India and will provide insights into gaps in the evidence base.

## Data Availability

No underlying data is associated with this article. The study will be reported using the Preferred Reporting Items for Systematic Reviews and Meta Analyses extension for Scoping Reviews (PRISMA-ScR) guidelines.
