# Supplementary material for: Overview of multimorbidity research in India: a scoping review protocol
Source: Wellcome Open Res. 2024 Dec 16;9:302. Originally published 2024 Jun 11. [Version 2] doi: 10.12688/wellcomeopenres.21378.2 (PMC11740011; doi:10.12688/wellcomeopenres.21378.2)
Supplement: Peer review history

# Reviewer report: Reviewer response for version 2

**DOI:** 10.21956/wellcomeopenres.25960.r116427

**Published:** 2025-01-15

**Authors:** Chris McParland

**Affiliations:** 1 University of Glasgow, Scotland, UK

This is an appropriately designed and well articulated protocol for a scoping review of multimorbidity research in India. The authors have provided a satisfactory response to prior reviewers, and adapted the protocol accordingly. As such, I have no reservations with its approval.

 I have one recommendation which the authors can use if helpful. In the search strategy, I would suggest broadening the synonyms for multimorbidity to include terms like pluripathology and polypathology. These are predominantly used in Spanish-speaking countries but occasionally feature in English-language publications. 

Is the study design appropriate for the research question?

Yes

Is the rationale for, and objectives of, the study clearly described?

Yes

Are sufficient details of the methods provided to allow replication by others?

Yes

Are the datasets clearly presented in a useable and accessible format?

Not applicable

Reviewer Expertise:

multimorbidity

I confirm that I have read this submission and believe that I have an appropriate level of expertise to confirm that it is of an acceptable scientific standard.

# Reviewer report: Reviewer response for version 2

**DOI:** 10.21956/wellcomeopenres.25960.r115739

**Published:** 2025-01-07

**Authors:** Josefien van Olmen

**Affiliations:** 1 University of Antwerp, Antwerp, Belgium

My comments have been adequately addressed

Is the study design appropriate for the research question?

Yes

Is the rationale for, and objectives of, the study clearly described?

Yes

Are sufficient details of the methods provided to allow replication by others?

Partly

Are the datasets clearly presented in a useable and accessible format?

Not applicable

Reviewer Expertise:

chronic diseases

I confirm that I have read this submission and believe that I have an appropriate level of expertise to confirm that it is of an acceptable scientific standard.

# Reviewer report: Reviewer response for version 2

**DOI:** 10.21956/wellcomeopenres.25960.r115738

**Published:** 2025-01-03

**Authors:** Susan Smith

**Affiliations:** 1 Trinity College Dublin, Dublin, Ireland

The authors have addressed my previous comments

Is the study design appropriate for the research question?

Yes

Is the rationale for, and objectives of, the study clearly described?

Partly

Are sufficient details of the methods provided to allow replication by others?

Partly

Are the datasets clearly presented in a useable and accessible format?

Not applicable

Reviewer Expertise:

Primary care, General Practice, Multimorbidity, Evidence Synthesis

I confirm that I have read this submission and believe that I have an appropriate level of expertise to confirm that it is of an acceptable scientific standard.

# Reviewer report: Reviewer response for version 1

**DOI:** 10.21956/wellcomeopenres.23643.r102330

**Published:** 2024-10-18

**Authors:** Susan Smith

**Affiliations:** 1 Trinity College Dublin, Dublin, Ireland

Thank you for asking me to review this proposal which outlines a protocol for a scoping review of research in multi morbidity has been conducted in India. Overall, it is well written and covers all aspects expected in a protocol. However, there are a number of areas that require further clarification.

 In particular, the distinction between multi morbidity and comorbidity needs to be considered clearly both in terms of these definitions and has a distinction in included studies will be addressed in the review. There is substantial literature outlining the difference between these two definitions (see https://journals.sagepub.com/doi/10.1177/2633556521993993 ). The main distinction is that studies addressing comorbidity will include an index condition, which means that all included participants will have that condition. Whereas, in multimorbidity studies there is no index condition and there can be multiple combinations of conditions depending on inclusion criteria.

 There is a significant amount of literature addressing the association between socio-economic status and multi morbidity and this could be considered both in the background and in terms of data extraction and interpretation.

 While the focus is very clearly on India, many of the issues that will be identified are also international problems and may be worth noting that.

**Methods**

**Objectives: **Given this is a scoping review and appropriate to include a broad range of study designs. However there is minimal focus on how distinctions between study designs will be addressed and reported. If interventional studies are identified, it would add value to explore their potential impact and this could be included as an additional secondary objective.

 Population - needs to be characterised as having multimorbidity. Also, what is morbidity in children it’s quite different to that an adults. This needs to be considered and reported separately.

**Concepts: **The term multimorbidity was in use well before 2018 - it only became an official MeSH term then.

**Exclusion criteria:** Give example here of the types of studies that might be excluded based on not having a focus on multimorbidity, eg focus on elderly or frailty without consideration or reporting of multimorbidity

**Table 2:** This is comprehensive in terms of outlining proposed data extraction but may need to also consider the following:

- Population characteristics: socioeconomic groups, ethnicity?
- Multimorbidity measurement: Types of condition in terms of acute vs chronic. Defined how? eg cancer
- Disease profiles. This level of detail may be very challenging to report
- Additional information that would be important to collect includes details of interventions, funding sources

Is the study design appropriate for the research question?

Yes

Is the rationale for, and objectives of, the study clearly described?

Partly

Are sufficient details of the methods provided to allow replication by others?

Partly

Are the datasets clearly presented in a useable and accessible format?

Not applicable

Reviewer Expertise:

Primary care, General Practice, Multimorbidity, Evidence Synthesis

I confirm that I have read this submission and believe that I have an appropriate level of expertise to confirm that it is of an acceptable scientific standard, however I have significant reservations, as outlined above.

# Response

**Published:** 2024-12-12

**Authors:** Parul Puri

**Affiliations:** International Institute for Population Sciences, India

**Comment:** **In particular, the distinction between multi morbidity and comorbidity needs to be considered clearly both in terms of these definitions and has a distinction in included studies will be addressed in the review. There is substantial literature outlining the difference between these two definitions (see https://journals.sagepub.com/doi/10.1177/2633556521993993 ). The main distinction is that studies addressing comorbidity will include an index condition, which means that all included participants will have that condition. Whereas, in multimorbidity studies there is no index condition and there can be multiple combinations of conditions depending on inclusion criteria.**

**Response** We thank the reviewer for the suggestions. We have now included this point in Background section. “Even though the terms multimorbidity and comorbidity are used interchangeably, they are related but different concepts – while both refer to people living with multiple chronic conditions, comorbidity refers to conditions in addition to an index condition, while multimorbidity is co-occurrence of two or more chronic conditions with no condition being prioritised over any other. ^1^ ^, 2^” In Methods section we have clarified that studies in India on comorbidity, that is ones examining co-occurring multiple long-term conditions in people living with an index condition, will be included in our scoping review. “All studies with a primary focus on multimorbidity will be included. Although the term ‘multimorbidity’ has been in use since 1976, more recently the definition has been revised and the distinction between comorbidity and multimorbidity has been highlighted. ^1,2^ We will include studies on comorbidity, that is ones examining co-occurring multiple long-term conditions in people living with an index condition and hence will be using both the terms ‘multimorbidity’ and ‘comorbidity’ during search.”  

**Comment:** **There is a significant amount of literature addressing the association between socio-economic status and multi morbidity and this could be considered both in the background and in terms of data extraction and interpretation.**

**Response** We agree with the reviewer that there is a substantial literature on the association between socio-economic status and multimorbidity. We have specified in our objectives that we will gather evidence on how risk factors for multimorbidity have been studied and have included socioeconomic status as one of the risk factors. In data extraction we have specified socioeconomic status as one the data points in the correlates domain.

**Comment** **While the focus is very clearly on India, many of the issues that will be identified are also international problems and may be worth noting that.**

**Response** Thank you for the suggestion. We have noted this in Background. “While the focus of this scoping review is on India, many of the topics discussed here may be applicable for other LMICs and for subpopulations in high-income countries.”

**Comment** **Methods**

**Objectives: Given this is a scoping review and appropriate to include a broad range of study designs. However there is minimal focus on how distinctions between study designs will be addressed and reported. If interventional studies are identified, it would add value to explore their potential impact and this could be included as an additional secondary objective.**

**Response** We agree with the reviewer that we should include studies with all study designs. We have specified in Population subsection that interventional studies will be included along with observational studies. However, based on our reading of the literature, we do not expect to find interventional studies on multimorbidity in India. Therefore, we have neither included this as one of the objectives, nor included any data item related to description of intervention in data extraction form. If interventional studies are found during search, we will extract information pertinent to the objectives we have specified. “All peer-reviewed, original full-text research of any study design (observational including cross-sectional and cohort, and interventional) with participants in any age range from India or its states/union territories, having multiple long-term conditions, will be included. ……….An initial search suggested multimorbidity studies looking at interventions may not yet exist in India. Hence, they are not included in objectives and are outside the scope of this review. However, if interventional studies are identified, data relevant to the stated objectives will be extracted.”  

**Comment** **Population - needs to be characterised as having multimorbidity. Also, what is morbidity in children it’s quite different to that an adults. This needs to be considered and reported separately.**

**Response** We have incorporated these suggestions while defining Population in Methods section. “All peer-reviewed, original full-text research of any study design (observational including cross-sectional and cohort, and interventional) with participants in any age range from India or its states/union territories, having multiple long-term conditions, will be included. Both children and adults will be considered and information on multimorbidity in children – conceptualization, definition, measurement, risk factors and outcomes – will be sought from studies including children and will be reported separately.”  

**Comment** **Concepts: The term multimorbidity was in use well before 2018 - it only became an official MeSH term then.**

**Response** Thank you for pointing this out. We have revised this paragraph. “All studies with a primary focus on multimorbidity will be included. Although the term ‘multimorbidity’ has been in use since 1976, more recently the definition has been revised and the distinction between comorbidity and multimorbidity has been highlighted. ^1,2^ We want to include studies on comorbidity, that is ones examining co-occurring multiple long-term conditions in people living with an index condition and hence will be using both the terms ‘multimorbidity’ and ‘comorbidity’.  We will also include similar terms like ‘polymorbidity’, 'multiple long-term condition(s), 'multiple chronic condition(s), 'multiple chronic disease(s)’ and 'multiple condition(s)'.”

**Comment** **Exclusion criteria: Give example here of the types of studies that might be excluded based on not having a focus on multimorbidity, eg focus on elderly or frailty without consideration or reporting of multimorbidity**

**Response** Per the suggestion, we have now included examples of studies that we will exclude. “We will exclude the following studies:

- Studies where multimorbidity is not the central theme, for example, focus is on elderly population or frailty without consideration or reporting of multimorbidity or multimorbidity is simply reported as one of the risk factors without providing any details regarding its definition or measurement.
- Studies examining only few, selected diseases or specific combinations such as studies of people with diabetes, hypertension and depression.
**Comment** **Table 2: This is comprehensive in terms of outlining proposed data extraction but may need to also consider the following:**
- **Population characteristics: socioeconomic groups, ethnicity?**
- **Multimorbidity measurement: Types of condition in terms of acute vs chronic. Defined how? eg cancer**
- **Disease profiles. This level of detail may be very challenging to report**
- **Additional information that would be important to collect includes details of interventions, funding sources **
**Response**
- We have made the following changes to data extraction table
- We have now clearly stated these factors in Study Participants and Characteristics
- We have now added this point when describing the type of conditions included - “How is chronicity defined and operationalized? How is the distinction made between chronic and acute conditions?”
- We agree with the reviewer that extracting and synthesizing this information can be difficult. We will focus on most prevalent combinations  and will report if there are common disease profiles across studies.
- As we have explained, interventions are outside the scope of the current review. Funding sources are important, but given that majority of the studies are secondary analyses of available data, we are unsure if this will be very helpful.

# Reviewer report: Reviewer response for version 1

**DOI:** 10.21956/wellcomeopenres.23643.r90357

**Published:** 2024-08-06

**Authors:** Josefien van Olmen

**Affiliations:** 1 University of Antwerp, Antwerp, Belgium

Rationale: They hint to other MM studies from other LMIC. But what is the current state of information about  definition, measurement tools and  analysis: a more specific overview of current findings makes it better possible to identify the specific added value of work. The Indian setting itself is not a justification, unless authors provide a reason why Indian-specific measures are necessary.

 Table 1 only comprises 4 cells. I presume the authors wil expand this. This needs to be done.

Is the study design appropriate for the research question?

Yes

Is the rationale for, and objectives of, the study clearly described?

Yes

Are sufficient details of the methods provided to allow replication by others?

Partly

Are the datasets clearly presented in a useable and accessible format?

Not applicable

Reviewer Expertise:

chronic diseases

I confirm that I have read this submission and believe that I have an appropriate level of expertise to confirm that it is of an acceptable scientific standard, however I have significant reservations, as outlined above.

# Response

**Published:** 2024-12-12

**Authors:** Parul Puri

**Affiliations:** International Institute for Population Sciences, India

**Comment:** **Rationale: They hint to other MM studies from other LMIC. But what is the current state of information about  definition, measurement tools and  analysis: a more specific overview of current findings makes it better possible to identify the specific added value of work. The Indian setting itself is not a justification, unless authors provide a reason why Indian-specific measures are necessary.**

**Response:** Thank you for pointing this out. We have expanded on this point in the Background section. “There are systematic reviews that have examined multimorbidity in low- and middle-income countries (LMICs) ^8^^– ^^12^ . In these reviews, the focus is on providing a burden estimate and describing patterns of occurrence. Although, different definitions, conditions included in the definition, and data sources are discussed to explain the heterogeneity in estimates and observed patterns, the reviews do not include details about types of conditions included, rationale behind inclusion and exclusion of conditions, how conditions are defined and measured and if any theoretical framework has been used to study risk factors and outcomes. Also, it is not clear if different data sources such as prescriptions, hospital discharge summary, insurance billing data, and lab investigations have been used in addition to diagnoses to study multimorbidity. These challenges, although common among LMICs, may vary between countries at different stages of multimorbidity research and data maturity. To understand how multimorbidity research in India can be strengthened, evidence specific to India is important. However, from the reviews in LMICs it is either difficult to gather information specific to India or the information is not comprehensive or up to date. A scoping review focusing on multimorbidity research in India will be able to fill this gap by systematically mapping the available evidence, thereby facilitating more robust and comparable findings in the future.”  

**Comment:** **Table 1 only comprises 4 cells. I presume the authors wil expand this. This needs to be done.**

**Response:** Yes, in the scoping review, we will provide the exact search strategy that was used for each of the databases. Here, we simply describe the principle.
